# Efficacy of Long-term Effect and Repeat Intraarticular Botulinum toxin in Patients with Painful Total Joint Arthroplasty: A Retrospective Study

**DOI:** 10.9734/BJMMR/2014/4897

**Published:** 2014-01-01

**Authors:** Jasvinder A. Singh

**Affiliations:** 1 Medicine Service and Center for Surgical Medical Acute Care Research and Transitions (C-SMART), Birmingham VA Medical Center, Birmingham, AL; USA; 2 Department of Medicine, University of Alabama, and Division of Epidemiology, School of Public Health, University of Alabama at Birmingham, Birmingham, AL; USA.; 3 Department of Orthopedic Surgery, Mayo Clinic School of Medicine, Rochester, MN, USA

**Keywords:** Intraarticular botulinum toxin, total joint arthroplasty, pain function

## Abstract

**Objective:**

Based on recent success of intra-articular (IA) Botulinum neurotoxin type A (BoNT/A; OnabotulinumtoxinA) in patients with osteoarthritis, we examined if repeat IA-BoNT/A is an effective antinociceptive in patients with refractory arthroplasty pain.

**Methods:**

11 patients with refractory chronic arthroplasty joint pain without any evidence of infection or prosthesis loosening were referred by orthopedic surgeons. After discussion of off-label use, each patient underwent IA injection of BoNT/A, repeated based on return of pain on numeric rating scale (NRS) and functional limitation on composite validated scales, Western Ontario McMaster Osteoarthritis index (WOMAC) or Shoulder Pain and Disability (SPADI).

**Results:**

11 patients (10 men, 1 woman) with 14 painful arthroplasty joints (3 bilateral; 12 knee and 2 shoulder) underwent ≥1 IA-BoNT/A injections (8 joints injected once, one joint injected twice only, five joints injected thrice) with doses ranging 100–300 units. Mean age was 68 years (standard deviation, 12) and follow-up ranged 1–28 months. Clinically meaningful reduction of 2-units in pain severity and really meaningful reduction in pain severity (50% reduction) were reported by 6/11 patients (6/13 joints) and 3/11 patients (3/13 joints), respectively, 1 month after the first IA-BoNT/A (100-units each). Significant improvements were noted in composite functional scales (WOMAC/SPADI). Pain relief was sustained at 3–4 month follow-up and was reproducible in those who received repeat injections. No significant adverse events were seen in any patients.

**Conclusions:**

A single intra-articular injection of BoNT/A improved pain and function in patients with chronic, refractory painful knee or shoulder arthroplasty, which sustained with repeat injections. Patients who were refractory to the first injection, did not respond to subsequent injections of higher dose of IA-BoNT/A.

## 1. INTRODUCTION

Total knee arthroplasty (TKA) is the commonest of all total Joint arthroplasty (TJA), with an annual volume of 300,000 in the U.S. in 2005 which is projected to increase 6-fold to 3.48 million/year by 2030 [1]. Total shoulder arthroplasty (TSA) is the third most common TJA (following knee and hip) that is becoming increasingly common. While most TJAs lead to significant improvement in pain, function and health-related quality of life (HRQoL), up to 8% of patients (40,000–65,000 patients/year in the U.S.) report persistence of moderate to severe index joint pain after arthroplasty [2]. Limited options exist for the treatment of chronically painful arthroplasty.

Botulinum neurotoxin type A (BoNT/A) is a neurotoxin approved for the treatment of several conditions including wrinkles, cervical dystonia etc. It can modulate the release of neuropeptides such as Substance P (SP) and calcitonin gene-related protein (CGRP) and inhibit neurogenic inflammation, [3] which likely underlies its independent antinociceptive effect. SP and CGRP may play a role in post-arthroplasty pain. The bone-prosthesis interface membranes obtained during revision surgery for painful primary hip arthroplasty had nerve fibers with positive immunostaining to SP, CGRP and Neurokinin A [4]. Joint fluid SP levels were elevated in painful knee joints with osteoarthritis that underwent TKA, but not in normal/asymptomatic contralateral knees [5]. The short-term antinociceptive action of IA-BoNT/A is supported by RCTs in patients with refractory shoulder[6], knee joint pain [7, 8] or painful arthroplasty[9]. However, long-term efficacy of IA-BoNT/A for painful TJA is not known, and it is not known if repeat injections are effective. We aimed to examine the long-term effects of IA-BoNT/A (OnabotulinumtoxinA) in patients with chronically painful TJA and the efficacy of repeat IA-BoNT/A injections in these patients.

## 2. METHODS

Using a retrospective study design, we reviewed prospectively collected clinical pain and function data on a consecutive sample of 11 patients with 14 painful TJA joints. The study was approved by the Minneapolis Veterans Affairs Medical Center’s Institutional Review Board. All patients were referred to the rheumatology clinic by orthopedic surgeons for potential novel therapies including IA-BoNT/A injection. The referring orthopedic surgeons worked up these patients with moderate to severe refractory prosthetic joint pain and ruled out implant infection, loosening or any surgically explainable cause of pain and determined that there were no surgically correctable causes for refractory arthroplasty pain. All patients had failed treatment with oral medications and were deemed as not candidates for revision arthroplasty. We provided a detailed explanation of “off label” use of IA-BoNT/A injection (not approved by the U.S. Food and Drug Administration for intra-articular use), known potential adverse effects as well as the potential risks of IA injection into a prosthetic joint. All subjects gave written informed consent for intraarticular injection and received a single IA-BoNT/A of 100 units (OnabotulinumtoxinA; BOTOX®, Allergan, Inc, Irvine CA) reconstituted in 5 ml of normal saline initially. Patients requested and received reinjection with IA-BoNT/A (100–300 units BOTOX®; reconstituted in 5 ml of normal saline) when the pain in the index joint returned to near pre-injection levels. If a patient had responded well to 100 units IA-BoNT/A, they received the subsequent injection with the same dose. If they had had no response, the dose was increased for the next injection. An accurate intra-articular needle placement was confirmed by aspiration of joint fluid in each case before injection. Patients were requested to return to the clinic every 1–3 months for a clinical follow-up. There was no placebo control and patients or providers were not blinded.

A standardized chart abstraction form was used to extract prospectively collected demographic data, treatments, pain severity, function assessment with Western Ontario McMaster Osteoarthritis index (WOMAC), Shoulder Pain and Disability (SPADI) and Timed Stands Test (TST), standard manual joint examination and possible adverse effects. WOMAC and TST were selected since they provide subjective and objective assessments of lower extremity function and pain; SPADI provides pain and function assessment for the shoulder. Pain severity was assessed on a 0–10 numeric rating scale (NRS) where 0 is ‘no pain’ and 10 is ‘worst possible pain’. Function was assessed by SPADI total score for shoulder [10] and WOMAC total score and TST for knees. A 2-point change or a change of 30% or greater on pain NRS is considered a clinically important change [11]; 50% change is considered really important change [12]. Lower extremity muscle strength was tested using manual muscle strength testing (grades 0–5) at each visit as part of a protocol to monitor for possible side effects of off-label Botulinum toxin [13]; in addition common side effects including any neurological deficits were monitored. We performed comparisons in daytime NRS pain, night-time NRS pain, WOMAC total, SPADI total and TST from pre-injection to post-injection follow-up, by using the analysis of variance (ANOVA) for repeated measures. A p-value ≤0.05 was considered statistically significant.

No funding was obtained for this study. Data are available for sharing with approvals from the appropriate institutions, Ethics committees and Privacy Protection for data transfer.

## 3. RESULTS

Eleven patients (10 men, 1 woman) with 14 chronically painful (pain >1-year in all patients) arthroplasty joints (11 TKA in 9 patients -two bilateral; and 3 TSA in 2 patients – one bilateral) underwent one or more IA-BoNT/A injections with doses ranging 100–300 units. The first injections were with 100 units IA-BoNT/A each. Repeat injections were 100–300 units. Eight joints were injected once (100 units BOTOX® each), one joint injected twice (100 units BOTOX® twice), five joints injected thrice (300 units BOTOX® in two patients and 100 units BOTOX® in three patients; four refractory TKAs and one responder). Mean age was 68 years (standard deviation, 12), 90% were men and follow-up ranged 1–28 months (mean, 12.3 months).

### 3.1 Improvement with First IA BoNT-A Injections

**Figure 1:**
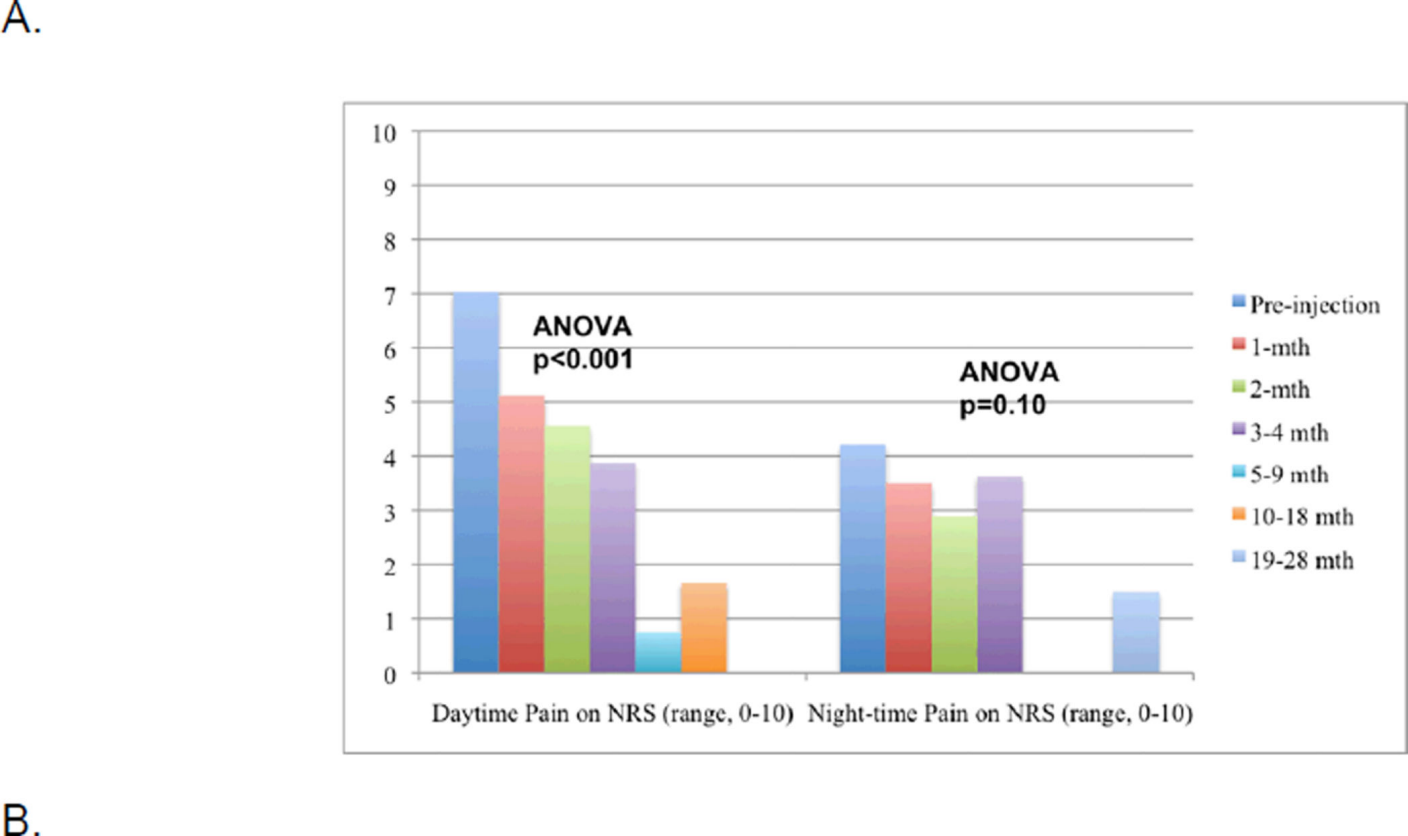

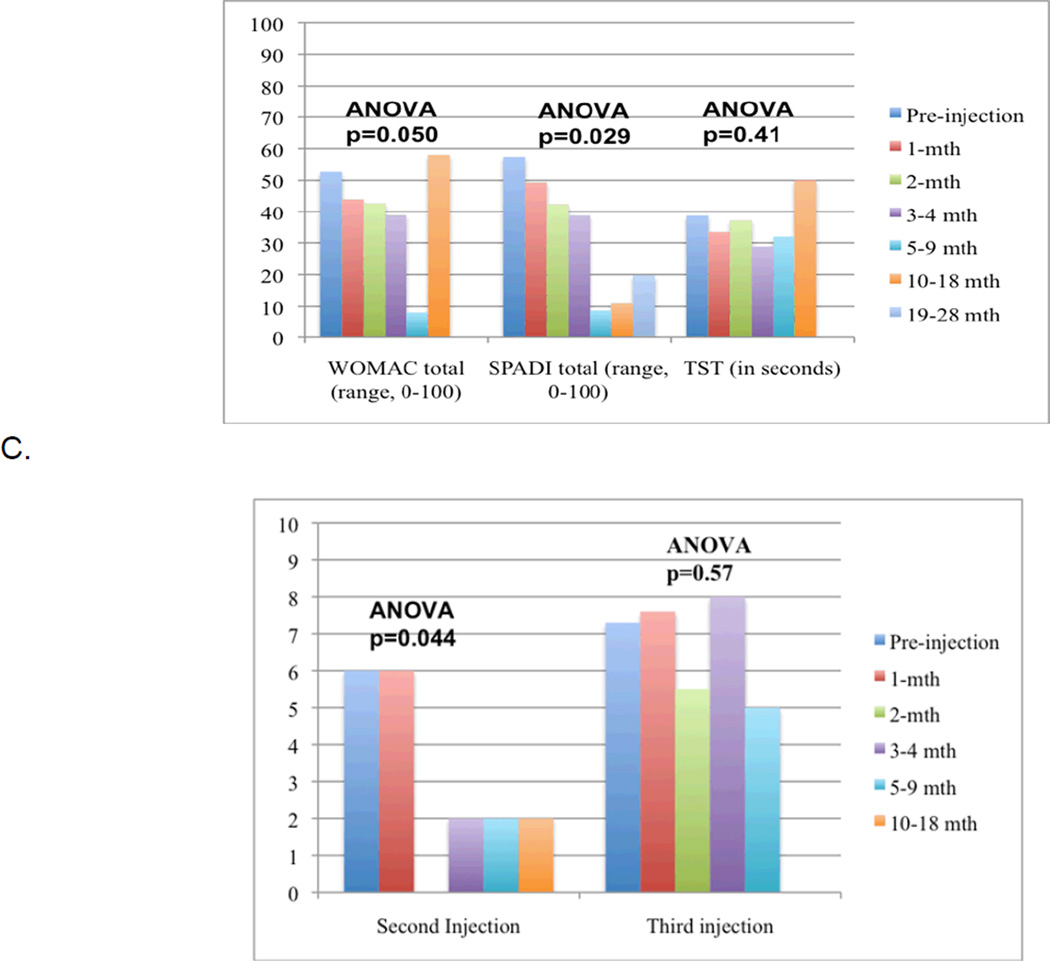
Change in 0–10 Numeric Rating Scale (NRS) Pain Intensity (1A) and validated lower and upper extremity function measures (WOMAC total score and Timed-Stands test for knee patients and SPADI total score for shoulder patients (1B) after first injection and Change in 0–10 NRS after subsequent injections (1C) 1A. Daytime and night-time Pain NRS (0–10) pre- and post-first IA-BoNT/A injection 1B. WOMAC total score (0–100), SPADI total score (0–100) and Timed Stands test (TST, in seconds) pre-post-first IA-BoNT/A injection 1C. Daytime NRS pain after second (n=6) and third (n=5) IA-BoNT/A injections X-axis represents time since the injection including the pre-injection time-point; in instances, when there are no observations at a specific time-point, the bar is missing. Y-axis represents pain severity from 0–10 numeric rating scale in figures 1A and 1C and the range for functional scale scores (WOMAC for knee and SPADI for shoulder) from 0–100 or timed stands test (TST) in seconds, for figure 1B. For example, in figure 1A, the first bar represents that daytime pain severity before the first IA BoNT-A injection and the next 5 bars daytime pain intensity upto 10–18 months after the first IA BoNT-A injection, showing significant reduction in pain severity from a mean of 7 pre-injection to <2 at the last follow-up (p<0.001 by ANOVA). Each patient received the first IA BoNT-A injection of 100 units. Similarly, figure 1C provides daytime pain NRS scores before (blue bars) and up to 10–18 months after second and third IA BoNT-A injections, respectively, showing that pain reduction was significant statistically for the second IA BoNT-A injection (p=0.044). IA BoNT-A doses were 100 units each in two joints and 200 units each in four joints. The pain reductions with third injections were not as impressive (p>0.05) and included two refractory patients, who had failed to respond to the first two injections. IA BoNT-A doses were 300 units in two joints and 100 units in three joints. Pain and functional scores were almost normally distributed with no skewed patterns.

Mean NRS daytime pain decreased significantly (p<0.001; Fig. 1A) from 7.0 (SD, 1.5) pre-injection to 5.1 (SD, 2.7) at 1-month after a single IA-BoNT-A injection of 100 units, with improvements sustained during the follow-up (Appendix 1). At 1-month post-injection, 54% reported clinically meaningful pain reduction (2-point) in NRS daytime pain and 27% reported 50% (really meaningful) pain reduction (Table 1). At 3–4 months after first IA-BoNT/A injection, 57% patients reported 50% reduction in NRS daytime pain (Table 1). Both patient-reported composite validated measures of lower and upper extremity function, the WOMAC total score (p=0.05) and SPADI total score (p=0.029), improved significantly after the injection (Fig. 1B). Night-time NRS pain showed a downward non-significant trend after IA-BoNT/A injection (p=0.10; Fig. 1A) and TST did not change significantly (p=0.41; Fig. 1B).

### 3.2 Repeat IA BoNT-A Injections

Four patients with six joints requested and received the second IA-BoNT/A injection (100 units each in two joints and 200 units each in four joints), which was associated with significant mean daytime NRS pain reduction (p=0.044), similar to the first injection (Fig. 1C). This cohort included two patients with prior good pain relief with first injection (responded again) and 2 patients with 4 TKAs with no prior pain relief (no response with second injection). Three patients with five joints received a third injection of IA-BoNT/A (2 patients with four refractory TKAs and one responder; 300 units in two joints and 100 units in three joints), with a non-significant improvement in mean daytime NRS pain (p=0.57) (Fig. 1C). No major adverse events were noted. Specifically, we did not observe recurrent falls or patient-reported paresthesias or muscle weakness or objective evidence of new extremity motor or sensory deficits, signs of joint inflammation, or septic arthritis, in any patient treated with IA BoNT-A.

## 4. DISCUSSION

This study provides evidence for efficacy of IA-BoNT/A in patients with refractory, chronically painful TJA not amenable to medical/surgical treatment, a difficult clinical problem. Our study provides a much longer follow-up compared to the 2-month primary outcome assessment in a randomized controlled trial. This study is the first time to provide the evidence related to the efficacy of repeat IA BoNT-A injections for the improvement of pain and function in patients with refractory, chronically painful TJA [9]. This observation also aligns well with previously observed sustained efficacy of IA-BoNT/A in refractory chronic arthritis joint pain [14]. Several patients had a sustained pain relief and improved patientreported extremity function after single injection of 100 units of IA-BoNT/A, with pain relief lasting as long as 28 months in one patient. Pain relief started as early as 1–2 months and reduction in pain was sustained at 3–4 and 5–9 month follow-up. A much smaller sample size at longer follow-up (>4-months) gives an impression of greater pain relief, but this represents a subsample of the original 11 patients and should be interpreted with caution. In our view, the pain relief started early and was sustained in these patients. We noted trend but not statistically significant improvement in night time pain, which may be due to lower preoperative night time pain score (floor effect, score of 4 compared to 7 for day time pain). Performance of TST requires good cardiopulmonary health and pre-injection TST had a large standard deviation, both of which may explain the absence of any difference on this objective measure of function after IA-BoNT/A.

These patients were deemed as not candidates for revision surgery by the referring orthopedic surgeons. While one needs to be very cautious regarding injecting a sterile prosthetic joint due to the potential risk of septic arthritis, in absence of an effective non-surgical treatment option, many of these patients seek and receive revision surgery (sometimes several procedures) for their refractory painful TJA, which clearly have higher infection risk and morbidity compared to an intraarticular injection. The beneficial effect of the first IA-BoNT/A injection was reproducible with repeat injections in several patients, without any evidence of loss of magnitude or duration of pain relief. BoNT has not been approved by the U.S. Food and Drug Administration for joint pain. Therefore, this constitutes an “off-label” use and should be considered only after a full understanding of risks/benefits by the patients and care providers. To our knowledge, this is one of the largest case series of IA therapy in patients with chronic, refractory, painful TJA.

Two patients with 4 painful TKAs did not respond to any dose of IA-BoNT/A, despite escalation of dose and three injections. That not all refractory painful TKAs will respond to IA-BoNT/A is an important observation. Most patients responded very well to IA-BoNT/A. This implies that among patients with chronic refractory painful TKA, most responded to 100 units initial dose of IA-BoNT/A, and that there was not a dose-threshold for antinociceptive effect. Due to a small number of patients, we were unable to determine as to what factors can predict the probability of response/non-response. No falls, neurological deficits, septic arthritis or systemic side effects of BoNT/A were noted in our small study.

Botulinum toxin A (BoNT/A) injections have an independent antinociceptive effect [15], in addition to the well-known anticholinergic effect (responsible for muscle-paralyzing action). This dual action was noted in cervical dystonia [16] and headache studies [17]. The antinociceptive effect is likely due to inhibition of neurogenic inflammation, [3] which is mediated by CGRP and substance P and blockade of local glutamate release that leads to local edema [18]. A recent systematic review summarized evidence from RCTs that supports the antinociceptive effect of BoNT/A in osteoarticular pain including patients with tennis elbow, low back pain, temporomandibular joint pain, carpal tunnel syndrome and plantar fasciitis [19].

Our retrospective study has several limitations. Due to the lack of blinding and placebo, results may be biased by patient expectation and patient/observer bias. However, all patients had failed 1–2 surgeries and multiple pain medications for persistent post-arthroplasty index joint pain. Pain assessments were done at clinic follow-up, which led to range of follow-up periods for this study, rather than fixed time-points in large randomized trials. The length of follow-up varied, since some patients did not return for follow-up after 6–12 months and several requested reinjections at 3–6 months when the effect of initial injection wore off. An exception was a patient who reported 100% pain relief up to 28 months after single injections in both index arthroplasty joints. These results may only be applicable to patients with chronic, unexplained, refractory prosthetic joint pain without infection, fracture or loosening/wear. We included several different doses and heterogeneous patient populations (previous responders in whom effect wore off and non-responders) for the second and third injections that likely impacted the results, therefore these results should be interpreted with caution. A small number of patients in our study did not allow us to observe any differences in patient characteristics between responders and non-responders. Our sample was predominantly men therefore these results may not be generalizable to women with painful, refractory TKA.

## 5. CONCLUSION

In conclusion, this small study adds to the growing evidence supporting the anti-nociceptive effects of intra-articular botulinum toxin and its potential benefit in patients with refractory, chronic post-prosthetic joint pain. This is a challenging clinical problem for reconstructive orthopaedic surgeons. Our study was limited in generalizability, had small numbers, was retrospective and unblinded. Nevertheless, it showed evidence of potential benefit in pain relief and functional improvement in patients with chronic unresponsive post-prosthetic joint pain. Thus, it intra-articular botulinum toxin might be a potential clinical tool for providers who take care of such patients. We recommend further investigations be undertaken to further validate our findings and more closely assess the potential benefits and harms of this treatment.

## Figures and Tables

**Table 1:** Clinically meaningful and Really Important changes in daytime NRS pain during follow-up after the first IA-BoNT/A injection

	2-point reduction (clinically meaningful change) % (n/N)		50% reduction (really important change) % (n/N)	

	Patients^a^	Joints	Patients	Joints
1-month post-injection	6/11 (54%)	6/13 (46%)	3/11 (27%)	3/13 (23%)
2-month post-injection	4/9 (44%)	4/11 (36%)	3/9 (33%)	3/11 (27%)
3–4 month post-injection	4/7 (57%)	5/9 (56%)	4/7 (57%)	4/9 (44%)
5–9 month post-injection	3/3 (100%)	4/4 (100%)	3/3 (100%)	4/4 (100%)
10–18 month post-injection	2/2 (100%)	3/3 (100%)	1/2 (50%)	2/3 (67%)
19–28 month post-injection	1/1 (100%)	2/2 (100%)	1/1 (100%)	2/2 (100%)

a One Patient missed the 1-month visit and did not provide follow-up pain data at that time

## APPENDIX

**Appendix 1:** Pre-and post-injection pain scores for the first, second and third IA-botulinum toxin injection

		First Injection			Second Injection			Third Injection		
	
	Time	N	Mean	SE	N	Mean	SE	N	Mean	SE
**Daytime Pain**	Pre-injection	14	7.0	0.4	6	6.3	0.4	5	7.3	0.4
	1-mth	13	5.1	0.7	6	5.9	0.9	5	7.6	0.9
	2-mth	9	4.5	0.9				2	5.5	2.5
	3–4mth	8	3.8	0.9	2	4.5	2.5	1	8.0	-
	5–9 mth	4	0.7	0.4	2	2.0	-	1	5.0	-
	10–18 mth	3	1.6	1.6	2	1.5	-			
	19–28 mth	1	0	-						
**Nighttime Pain**	Pre-injection	14	4.2	0.7	6	4.3	1.0	5	2.8	1.0
	1-mth	12	3.5	0.8	6	3.5	1.2	5	3.8	1.2
	2-mth	9	2.8	0.9				2	3.0	0
	3–4mth	8	3.6	1.2	2	3.5	0	1	3.0	-
	5–9 mth	4	0	0	2	4.0	-	1	3.0	-
	10–18 mth	3	0	0	2	0	-			
	19–28 mth	1	1.5	-						
**WOMAC total**	Pre-injection	11	52.7	3.4	6	55.0	1.3	5	61.8	3.1
	1-mth	11	44.0	5.6	6	53.0	7.7	5	61.8	6.7
	2-mth	6	42.6	8.9				2	49.0	27.0
	3–4mth	6	38.8	8.3	2	53.0	24.0	1	76.0	-
	5–9 mth	2	8.0	2.0	2	33.5	14.5	1	31.0	-
	10–18 mth	1	58.0	-	2	39.5	2.5			
	19–28 mth									
**SPADI total**	Pre-injection	3	21.5	10.0	1	0	-	1	0	-
	1-mth	3	16.4	11.2	1	0	-	1	0	-
	2-mth	3	25.4	11.4				1	0	-
	3–4mth	3	9.6	9.6	1	0	-	1	0	-
	5–9 mth	2	5.8	4.1	1	0	-	1	0	-
	10–18 mth	2	10.9	4.9						
	19–28 mth	2	19.7	-						
**TST**	Pre-injection	10	38.7	4.0	6	38.6	4.1	3	40.6	2.3
	1-mth	11	33.4	3.2	6	38.0	2.2	3	38.6	6.3
	2-mth	6	37.1	4.8				2	41.0	16.0
	3–4mth	6	28.8	3.0	2	30.0	7.0	1	57.0	-
	5–9 mth	1	32.0	-	1	32.0	-	1	27.0	-
	10–18 mth	1	50.0	-	2	27.0	0			
	19–28 mth									

-, not calculable, SE = standard error, TST, timed stands test, SPADI, Shoulder Pain and Disability Index, WOMAC, Western Ontario and McMaster Arthritis Index

Cells with missing for “N” are the ones with no observation for that time-point
